# Editorial: Precision control technology and application in agricultural pest and disease control

**DOI:** 10.3389/fpls.2023.1163839

**Published:** 2023-02-27

**Authors:** Yunchao Tang, Chao Chen, Antonio Candea Leite, Ya Xiong

**Affiliations:** ^1^ College of Urban and Rural Construction, Zhongkai University of Agriculture and Engineering, Guangzhou, China; ^2^ Department of Mechanical and Aerospace Engineering, Monash University, Melbourne, VIC, Australia; ^3^ Faculty of Science and Technology, Norwegian University of Life Science, Ås, Norway

**Keywords:** computer vision, digital precision monitoring, seedling protection, green pesticides, eco-friendly pest control technology, spraying system

## 1 Introduction

The Food and Agriculture Organization of the United Nations (FAO) reports that up to 40% of global crop production is lost annually due to weeds, pests, and diseases, and these losses could worsen without proper pest and disease management (OECD/FAO, 2012). Conventional approaches to pest monitoring and management are insufficient in meeting present demands in terms of efficiency, coverage, and cost-effectiveness. (Wolff et al., 2016). To address this issue, the development of smart pest control technology (Kanwal et al., 2022), the improvement of agricultural pest control systems, and stronger regulation of foreign species are demanded to collect pest outbreak data in a timely, accurate, and comprehensive manner. In the future, the focus of agricultural pest control will be on developing the fundamental theories, key technologies, and major products and equipment of “preventable,” “controllable,” “treatable,” and “green” pest control throughout the process.

## 2 Articles in this research topic

Given the need for precision pest and disease control, this special topic was organized which collected several research papers, covering pest and disease monitoring, detection and classification methods, as well as the development of control systems, with a strong focus on disease detection.

### 2.1 Detection and monitoring of plant pest and disease

Maize disease detection and classification has attracted great attention. Fu et al. proposed a maize spectral recovery disease detection framework based on HSCNN+ and maize disease detection CNN to complete low-cost and high-precision maize disease detection. They found an ideal spectral recovered model to reconstruct HSI data from the raw maize RGB data. Haque et al. presented a lightweight CNN model to classify different severity stages of Maydis leaf blight disease. Their results showed that the proposed model outperformed most of the commonly used pretrained models in terms of classification accuracy. Similarly, Albahli and Masood presented an end-to-end learning CNN architecture, namely EANet, to identify multi-class maize diseases. An attention mechanism was used to improve the performance of recognizing multiple diseases. Results showed that the proposed method was able to classify maize leaf diseases in complex background settings. Researchers also demonstrated pest and disease monitoring and recognition methods for rice crops and jujube trees. Li et al. developed an intelligent monitoring system to detect the disease and pest lesions on the rice canopy. They proposed an improved YOLO-DPD model to capture the features of rice disease and pest lesions. Wu et al. used hyperspectral remote sensing images from UAVs to recognize and monitor spider mite infestations in jujube trees. The recognition model combined spectral features and spatial information, and has performed well in the experiments.

### 2.2 Pest and disease control system and mechanisms

In addition to detection and monitoring, researchers also showed recent developments for pest and disease control system. Li et al. developed a real-time target spraying system based on an improved CNN model. Field tests demonstrated that their spraying system could accurately hit target weeds. Jiang et al. studied the wetting and deposition mechanism of spraying droplet. They found the best nozzle type, spray pressure and wind speed settings for spray coverage and droplet density.

## 3 Perspectives

The editorials suggest that further research and technology application should be conducted in the following areas:

### 3.1 Construction and application of a digital and precise monitoring and early warning technology system for agricultural, forestry, and grassland pest control

The editorial team suggests that we should focus on the lack of accurate monitoring and early warning technologies for major pests and diseases in agriculture, and develop core key technologies such as intelligent pest and disease identification and automatic counting technology, and machine learning-based image recognition technology. We should research and develop key technologies, core equipment, and imaging acquisition standards for ground-based camera capture, low-altitude unmanned aerial vehicle remote sensing, and high-altitude radar monitoring of pests and diseases, and establish a massive high-quality image database. We should also develop big data collection, multi-source information processing and transmission, and rapid monitoring and evaluation technologies based on the Internet of Things and cloud computing, and establish a nationwide database of agricultural and forestry pest and disease occurrence and their suitable environments. We should break through the large-scale intelligent identification and quantification extraction method based on machine vision, establish a multi-temporal comprehensive forecasting and warning model based on multi-source information on the occurrence and breeding dynamics of major pests and diseases, host growth conditions, and meteorological adaptation. Finally, we should develop an integrated and map-based analysis platform for real-time accurate monitoring, early warning and efficient information dissemination of emerging pest and disease outbreaks, and construct a spatiotemporal dynamic visualization and intelligent monitoring and warning system for pest and disease populations.

### 3.2 Mechanisms of signal communication between pest diseases and crops and new controlling-attracting techniques

The editorial team recommends research in four areas to develop new strategies and techniques for pest and disease control:

Firstly, researchers should investigate the mechanisms of signal communication during major pest and disease outbreaks in crops and analyze the information exchange between individual pests and diseases that lead to such outbreaks.

Secondly, researchers should elucidate the mechanisms of identification, decoding, and transmission of inter-species information flow between pests, diseases, and crops.

Thirdly, researchers should explore the defense responses and regulatory mechanisms of crops against pest and disease infestations.

Finally, to develop new strategies and techniques for pest and disease control, researchers should also actively develop high-throughput screening methods to identify chemical communication substances and their analogues, design target-releasing and enhancing agents, and research and develop pest and disease control products for field use based on the interactions between biological information flows.

### 3.3 Research and development of mechanisms and control technologies for important pests and diseases resistance in agriculture

The editorial team believes that in order to combat important pests and pathogens that pose a serious threat to major crops such as rice, corn, cotton, and vegetables worldwide, we need to conduct risk assessments and interactive resistance studies on these pests and their resistance to new drug agents. We need to analyze the molecular mechanisms of resistance formation from aspects such as target mutation and detoxification metabolism enhancement. Research is needed on the genetic diversity and adaptive evolution mechanisms of resistance genes. The causes and evolutionary laws of multidrug resistance should also be studied. Furthermore, we should develop resistance diagnosis, monitoring, warning, and control technologies based on key resistance genes.

### 3.4 Succession laws of pests diseases and the whole-process green control technology system

The editorial team proposes urgent research to address the major challenges presented by the catastrophic mechanisms and control techniques associated with serious pest and disease outbreaks, including the Yangtze River Delta beetle, canker disease, and other serious pests and diseases that pose a major threat to the safety of key protected forests worldwide. The effects of industrial structure adjustment and climate change on the succession patterns of major pests and diseases, such as diamondback moth, tobacco whitefly, root-knot nematode, and viral diseases, are examined to clarify their harmful rules and mechanisms. This includes investigating the adaptation, harmful variation, regional disasters, and periodic outbreak mechanisms of pests and diseases under changing climatic conditions; conducting health evaluations, precise monitoring, and big data forecasting of protective forests, and researching the autonomous pest resistance of main afforestation species and its induced enhancement techniques; integrating and optimizing the diverse and coordinated application technologies of different pest control technologies and products such as lures, natural enemies, microorganisms, and natural active substances; developing natural pest and disease control techniques that focus on forest optimization, nurturing, and renewal measures; and constructing integrated green pest and disease control technology schemes for multiple pest and disease types throughout the entire growth cycle.

### 3.5 Exploration of biocontrol microbial resources and their interaction mechanisms with plants

The editorial team points out the current shortage of important biological control products for pest and disease management. To address this issue, we need to focus on major crops such as grains, oilseeds, economic crops, and forest and grassland plants, and screen for highly efficient biocontrol microbial strains. We advocate for scholars to study the persistence and control mechanisms of biocontrol microbes, identify new functional genes of biocontrol strains, use modern biological breeding technology to modify production strains, enhance product biocontrol activity, broaden the spectrum of insecticidal and fungicidal activities, and improve stress resistance. We aim to overcome the three waste problems of biocontrol microbial fermentation, develop efficient green production processes and supporting equipment, create new formulations and products for important pests and diseases and corresponding application technologies that are suitable for the planting technology system. We aim to reveal the pathogenic mechanisms of microbial pathogens and plant immune defense mechanisms, develop new strategies and technologies for plant disease control, analyze the interactions among plants, pests, and natural enemies, and the impact mechanisms of multiple ecological factors such as microbes and the environment, and develop new green pest control technologies for agriculture and forestry.

## Author contributions

All authors listed have made a substantial, direct, and intellectual contribution to the work and approved it for publication.
